# Body roundness index and the risk of knee osteoarthritis: evidence from the China Health and Retirement Longitudinal Study

**DOI:** 10.3389/fnut.2025.1533966

**Published:** 2025-03-12

**Authors:** Zong Jiang, Xin Cai, Xiaoling Yao, Weiya Lan, Xueming Yao, Fang Tang, Wukai Ma

**Affiliations:** ^1^Second Clinical Medical College, Guizhou University of Traditional Chinese Medicine, Guiyang, China; ^2^Department of Rheumatology and Immunology, The First People's Hospital of Guiyang, Guiyang, China; ^3^Department of Rheumatology and Immunology, The Second Affiliated Hospital of Guizhou University of Traditional Chinese Medicine, Guiyang, China

**Keywords:** knee osteoarthritis, body roundness index, China Health and Retirement Longitudinal Study, longitudinal study, risk

## Abstract

**Objective:**

Previous cross-sectional studies have demonstrated that the body roundness index (BRI) is associated with knee osteoarthritis (KOA). However, no longitudinal studies have confirmed this association. This study aims to explore the link between BRI and KOA risk in the Chinese population through longitudinal analysis and to evaluate its utility in early diagnosis and risk prediction.

**Methods:**

This study utilizes data from the China Health and Retirement Longitudinal Study (CHARLS). A total of 7,318 participants who were followed from 2015 to 2020 were included. BRI was calculated using physical examinations and questionnaire data, and participants were categorized by quartiles. The relationship between BRI and KOA risk was assessed using multivariate weighted regression models and trend tests, while subgroup and sensitivity analyses were conducted to ensure the robustness of the findings.

**Results:**

After 5 years of follow-up, 1,035 participants (14.14%) were diagnosed with KOA. Findings indicate a positive correlation between BRI and KOA risk (*HR* = 1.08, 95% *CI*: 1.02–1.13, *p* = 0.0039), with an increasing trend in KOA risk across BRI quartiles (*p* for trend = 0.0033). Subgroup analysis reveals that the association is particularly strong among individuals aged 50–59, males, those living in rural areas, and those without cardiovascular disease.

**Conclusion:**

This study establishes that an increase in BRI significantly elevates KOA risk. These findings suggest that BRI could be an effective tool for KOA risk assessment and could contribute to the development of personalized prevention strategies. Additionally, BRI is valuable in elucidating the potential mechanisms linking body fat distribution and inflammatory responses in KOA progression.

## 1 Introduction

Osteoarthritis (OA) is the most common cause of joint pain worldwide, affecting all joints throughout the body, with knee osteoarthritis (KOA) being the most common clinical manifestation (1, 2). Its etiology is multifactorial, encompassing mechanical load, metabolic disorders, chronic inflammation, and genetic factors (3, 4). In addition, some risk factors such as age, female gender, and history of joint injuries are common causes of knee osteoarthritis [38588890]. The common pathogenic characteristics of these risk factors are synovitis and cartilage degeneration in the joint cavity, which induce the release of inflammatory factors and cause persistent joint pain (5), especially in the joint cavity where physical injuries such as meniscus cause synovitis and trigger the release of inflammatory chemokines, leading to a more obvious cascade reaction of inflammation (6). As the world's second most populous country, China is experiencing a rising prevalence of KOA, driven by an aging population and lifestyle changes, posing a significant public health challenge (7). A longitudinal study over 4 years reported a high cumulative incidence of symptomatic KOA among older adults in China at 8.5% (8). Despite this, effective markers for predicting KOA remain inadequate (9). With the growing issue of obesity, the link between obesity-related metrics and KOA has garnered increasing attention (10). Unlike the traditional body mass index (BMI), which is calculated from height and weight and often used to assess obesity and associated health risks, BMI does not capture variations in fat distribution and body shape, and its suitability for children and adolescents is limited (11). Consequently, there is a pressing need for more accurate and comprehensive tools to assess and predict health risks.

Body Roundness Index (BRI), introduced by Thomas et al., is an advanced anthropometric measure that effectively reflects abdominal fat accumulation and total body fat by incorporating the geometric characteristics of waist circumference and height (11). BRI is applicable across different genders and ages, demonstrates a stronger correlation with metabolic diseases, accounts for body shape, and offers enhanced risk stratification, addressing the limitations of BMI. These advantages have facilitated its widespread use in disease prevention (11). Prior research has established a strong association between BRI and metabolic syndrome, insulin resistance, and cardiovascular diseases (12–14), which are also key risk factors for the development of KOA (15, 16). Furthermore, obesity may significantly influence the pathogenesis of KOA through increased mechanical load and induction of low-grade inflammation (17). Nevertheless, studies investigating the link between BRI and KOA risk are scarce (18–20), these research reports are all from the National Health and Nutrition Examination Survey (NHANES), which confirms a positive correlation between BRI and OA. And these studies are all cross-sectional studies, lacking longitudinal cohort studies, especially in specific populations where longitudinal studies are almost non-existent. Consequently, a thorough examination of BRI's potential for early diagnosis and risk prediction of KOA is imperative.

The China Health and Retirement Longitudinal Study (CHARLS) has gathered high-quality microdata from individuals aged 45 and above across 28 provinces, 150 county-level units, and 450 village-level units in China. Its primary goal is to advance interdisciplinary research on aging by addressing the challenges of population aging (21, 22). Data collection in CHARLS is primarily conducted through questionnaire surveys, covering basic personal information, health status, physical measurements, employment, retirement, and pensions (21). Utilizing the CHARLS database, this study examines the relationship between BRI and KOA, aiming to establish a scientific foundation for early identification, precise prevention, and strategy development for KOA. It also seeks to provide new insights into BRI and its associated metrics in joint degenerative diseases.

## 2 Methods

### 2.1 Study design and population

This study adhered to the guidelines and recommendations of CHARLS. The dataset is available at http://charls.pku.edu.cn. We included participants from the 2015 survey who underwent physical examinations and provided baseline data. We have established some criteria to exclude participants who do not meet the research criteria: (1) those who were under 45 years old when participating in the questionnaire survey in 2015; (2) Diagnosed with KOA; (3) Lack of BRI survey information; (4) Lack of information on potential KOA influencing variables; (5) Participants who were lost to follow-up in 2020. As CHARLS received approval from the Institutional Review Board of Peking University and all participants provided written informed consent, no further ethical approval was required for this study.

### 2.2 BRI assessment

BRI was developed by Thomas et al. using three independent databases with 7166 participants and validated through variable analysis such as height, weight, race, and body fat percentage. The prediction formula was calculated by rigorously measuring waist circumference and height, as follows: BRI = 364.2–365.5 × [1 – (WC (m)/2π)^2^/(0.5 × height (m))^2^]^1/2^ (11). These data were collected by professionally trained personnel to ensure the reliability of the results.

### 2.3 KOA assessment

The diagnosis of KOA was determined based on the physical examination and questionnaire survey of CHARLS in 2015. Participants were asked: (1) “Has a doctor diagnosed you with arthritis/rheumatism?”; (2) “Are you often troubled by physical pain?” If the answer to both questions was “No,” KOA was excluded. If “Yes,” they proceeded to the next question: (3) For those who responded “Yes,” a card depicting a human figure with marked joints was presented, and participants were asked to identify the painful joints, including the knee. If knee pain was reported, either independently or in conjunction with a doctor's diagnosis of arthritis/rheumatism, KOA was confirmed (8).

### 2.4 Covariate assessment

To mitigate the influence of potential confounding factors, further analyses of covariates that might impact BRI and KOA risk were conducted. Based on the variables collected from the CHARLS database questionnaire survey, reference to previous research variables, and clinical experience selection, the following variable adjustments will be made (18), included: (1) Demographic data (Age, BMI, Sex, Education, Marital status, Residence); (2) Questionnaire data (Smoking, Drinking, Self-health, Life-satisfaction, Fall, Stroke, Diabetes, Cardiovascular Disease, Dyslipidemia, Lung disease); (3) Laboratory data [white blood cell (WBC); platelet (PLT), low-density lipoprotein cholesterol (LDL-C)].

### 2.5 Statistical analysis

Baseline characteristics of participants were presented as frequencies (%) for categorical variables and means ± standard deviations ($\bar{x}$ ± *s*) for continuous variables. Chi-square tests and either the Kruskal-Wallis H test or analysis of variance (ANOVA) were used depending on the normality of data distribution. Analyses utilized a weighting scheme, and results for continuous and categorical variables were reported as weighted means (95% CI) and proportions (95% CI), respectively. The relationship between BRI and KOA risk was examined using weighted multivariate regression analysis, with covariates adjusted separately to ensure the validity of the results. Three models were constructed: Model 1 assessed the basic association between BRI and KOA risk; Model 2 further adjusted for age, sex, education, marital status, and residence; Model 3 included comprehensive adjustments for demographic and health-related variables. The credibility of the regression results was verified through trend tests, and potential linear associations were evaluated using smooth curve fits and weighted generalized additive models. Subgroup analyses were conducted based on age, sex, residence, and health conditions to examine the relationship between BRI and KOA risk. Sensitivity analysis employed the receiver operating characteristic curve (ROC) comparing BRI with BMI to evaluate the predictive model's effectiveness. All statistical analyses were performed using SPSS 25.0 (IBM Corp., Armonk, NY, USA), R software (http://www.R-project.org), and EmpowerStats (http://www.empowerstats.com), and a *p* < 0.05 was deemed statistically significant.

## 3 Results

### 3.1 Characteristics of the study population based on BRI quartiles

**Figure 1:**
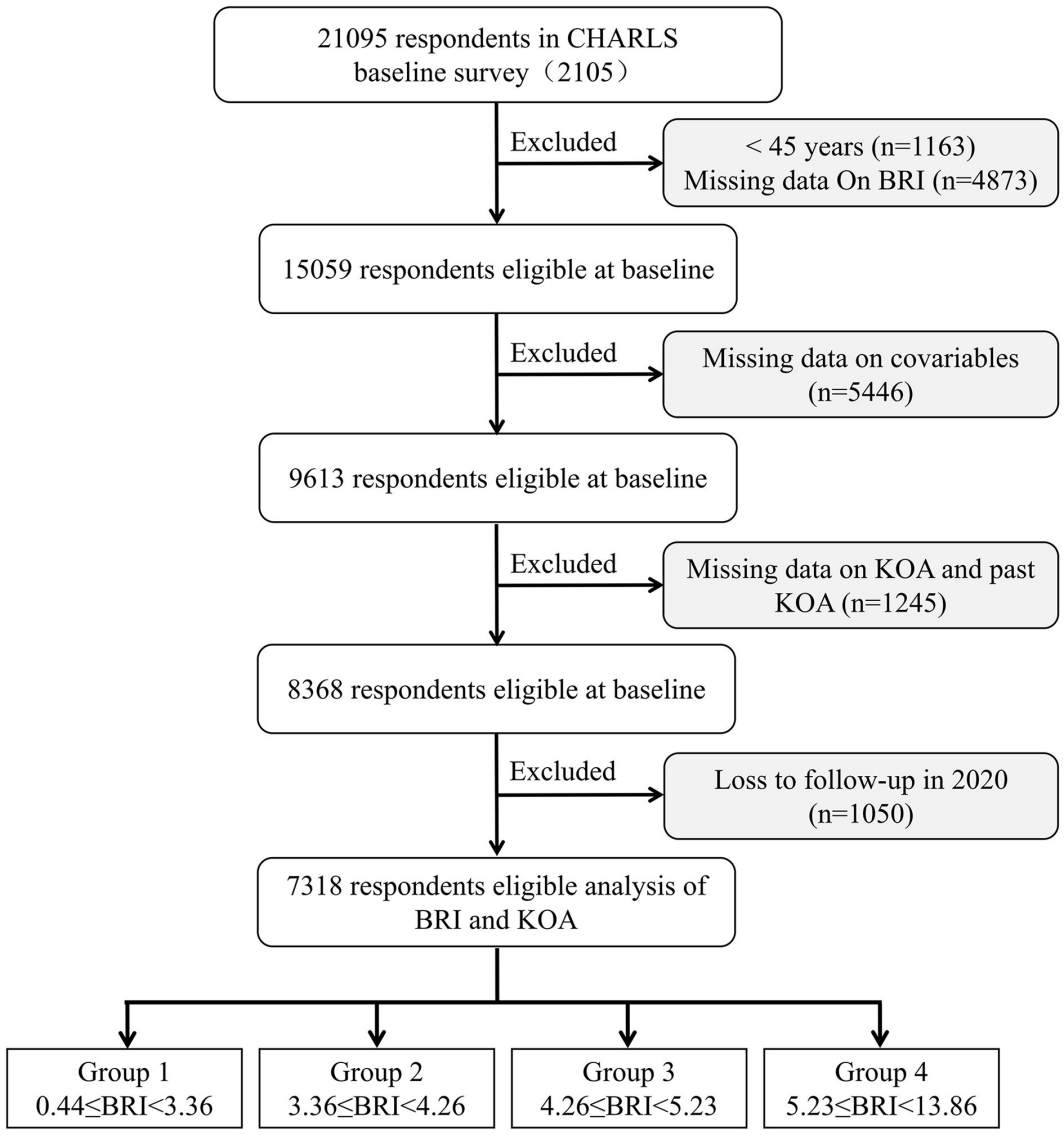
Flowchart of participants' selection. BRI, body roundness index; CHARLS, China Health and Retirement Longitudinal Study; KOA, knee osteoarthritis.

Following inclusion and exclusion criteria and subsequent follow-up, participants under 45 years, lacking BRI data, or previously diagnosed with KOA were excluded. Of the 21,095 participants surveyed in 2015, 1,163 under 45 and 4,873 with incomplete BRI data were excluded. To minimize confounding effects, additional covariate adjustments were made, excluding 5,446 participants lacking necessary covariates, and 1,245 diagnosed with KOA at the time of the 2015 survey. Additionally, 1,050 participants lost to follow-up by 2020 were also excluded. Ultimately, 7,318 participants were selected for analysis (Figure 1). The 7,318 participants (3,811 females and 3,507 males) with a median age of 60.76 years were included. At the 5-year follow-up, 1,035 individuals (14.14%) had developed KOA. After grouping by BRI quartiles, the incidence of KOA gradually increased (11.48 vs. 13.01 vs. 14.05 vs. 18.03, *p* < 0.001), with 0.44 ≤ Q1 < 3.36 (*n* = 1,830), 3.36 ≤ Q1 < 4.26 (*n* = 1,829), 4.26 ≤ Q1 < 5.23 (*n* = 1,830), and 5.23 ≤ Q1 ≤ 13.86 (*n* = 1,830). Significant differences were observed in terms of age, WBC, PLT, LDL-C, BMI, sex, education, marital, residence, smoking, drinking, self-health, life-satisfaction, diabetes, cardiovascular disease, dyslipidemia, and lung disease among the participants (all *p* < 0.05; Table 1).

**Table 1:** Characteristics of the study population according to BRI quartiles.

**BRI quartile**	**All**	**Q1 (0.44–3.36)**	**Q2 (3.36–4.26)**	**Q3 (4.26–5.23)**	**Q4 (5.23–13.86)**	***P*-value**
*N*	7,318	1,830	1,829	1,829	1,830	–
Age (years)	60.76 ± 8.76	61.07 ± 8.91	60.38 ± 8.68	60.28 ± 8.69	61.30 ± 8.74	< 0.001
WBC (1 × 10^9^/L)	5.94 ± 1.75	5.78 ± 1.92	5.83 ± 1.66	5.99 ± 1.68	6.16 ± 1.72	< 0.001
PLT (10^9^/L)	204.12 ± 72.88	194.53 ± 74.22	202.43 ± 68.83	208.03 ± 73.05	211.50 ± 74.19	< 0.001
LDL-C (mg/dl)	102.71 ± 28.97	96.98 ± 27.74	102.45 ± 28.07	104.97 ± 29.39	106.43 ± 29.74	< 0.001
BMI (kg/m^2^)	24.00 ± 3.72	20.31 ± 2.06	22.84 ± 1.93	24.85 ± 2.09	27.99 ± 3.38	< 0.001
**Sex (%)**						< 0.001
Female	3,811 (52.08%)	586 (32.02%)	868 (47.46%)	1,042 (56.97%)	1,315 (71.86%)	
Male	3,507 (47.92%)	1,244 (67.98%)	961 (52.54%)	787 (43.03%)	515 (28.14%)	
**Education (%)**						< 0.001
Primary school and below	3,016 (41.21%)	725 (39.62%)	689 (37.67%)	714 (39.04%)	888 (48.52%)	
Junior high school	1,690 (23.09%)	462 (25.25%)	433 (23.67%)	413 (22.58%)	382 (20.87%)	
Senior high school	1,724 (23.56%)	444 (24.26%)	460 (25.15%)	451 (24.66%)	369 (20.16%)	
University and above	888 (12.13%)	199 (10.87%)	247 (13.50%)	251 (13.72%)	191 (10.44%)	
**Marital (%)**						0.018
Unmarried	828 (11.31%)	201 (10.98%)	183 (10.01%)	202 (11.04%)	242 (13.22%)	
Married	6,490 (88.69%)	1,629 (89.02%)	1,646 (89.99%)	1,627 (88.96%)	1,588 (86.78%)	
**Residence (%)**						< 0.001
Urban	2,726 (37.25%)	550 (30.05%)	638 (34.88%)	762 (41.66%)	776 (42.40%)	
Rural	4,592 (62.75%)	1,280 (69.95%)	1,191 (65.12%)	1,067 (58.34%)	1,054 (57.60%)	
**Smoking (%)**						< 0.001
No	4,109 (56.15%)	698 (38.14%)	979 (53.53%)	1,122 (61.34%)	1,310 (71.58%)	
Yes	3,209 (43.85%)	1,132 (61.86%)	850 (46.47%)	707 (38.66%)	520 (28.42%)	
**Drinking (%)**						< 0.001
No	3,925 (53.63%)	826 (45.14%)	941 (51.45%)	976 (53.36%)	1,182 (64.59%)	
Yes	3,393 (46.37%)	1,004 (54.86%)	888 (48.55%)	853 (46.64%)	648 (35.41%)	
**Self-health (%)**						0.003
Excellent	221 (3.02%)	58 (3.17%)	50 (2.73%)	45 (2.46%)	68 (3.72%)	
Very good	1,064 (14.54%)	293 (16.01%)	243 (13.29%)	249 (13.61%)	279 (15.25%)	
Good	4,136 (56.52%)	1,038 (56.72%)	1,048 (57.30%)	1,006 (55.00%)	1,044 (57.05%)	
Fair	932 (12.74%)	234 (12.79%)	245 (13.40%)	255 (13.94%)	198 (10.82%)	
Poor	965 (13.19%)	207 (11.31%)	243 (13.29%)	274 (14.98%)	241 (13.17%)	
**Life-satisfaction (%)**						0.003
Completely satisfied	82 (1.12%)	21 (1.15%)	21 (1.15%)	22 (1.20%)	18 (0.98%)	
Very satisfied	394 (5.38%)	125 (6.83%)	93 (5.08%)	99 (5.41%)	77 (4.21%)	
Somewhat satisfied	3,582 (48.95%)	902 (49.29%)	880 (48.11%)	932 (50.96%)	868 (47.43%)	
Not very satisfied	2,786 (38.07%)	649 (35.46%)	713 (38.98%)	663 (36.25%)	761 (41.58%)	
Not at all satisfied	474 (6.48%)	133 (7.27%)	122 (6.67%)	113 (6.18%)	106 (5.79%)	
**Fall (%)**						0.299
No	6,216 (84.94%)	1,553 (84.86%)	1,566 (85.62%)	1,566 (85.62%)	1,531 (83.66%)	
Yes	1,102 (15.06%)	277 (15.14%)	263 (14.38%)	263 (14.38%)	299 (16.34%)	
**Stroke (%)**						0.196
No	7,121 (97.31%)	1,789 (97.76%)	1,784 (97.54%)	1,779 (97.27%)	1,769 (96.67%)	
Yes	197 (2.69%)	41 (2.24%)	45 (2.46%)	50 (2.73%)	61 (3.33%)	
**Diabetes (%)**						< 0.001
No	6,663 (91.05%)	1,755 (95.90%)	1,686 (92.18%)	1,657 (90.60%)	1,565 (85.52%)	
Yes	655 (8.95%)	75 (4.10%)	143 (7.82%)	172 (9.40%)	265 (14.48%)	
**Cardiovascular disease (%)**						< 0.001
No	6,200 (84.72%)	1,635 (89.34%)	1,580 (86.39%)	1,552 (84.86%)	1,433 (78.31%)	
Yes	1,118 (15.28%)	195 (10.66%)	249 (13.61%)	277 (15.14%)	397 (21.69%)	
**Dyslipidemia (%)**						< 0.001
No	5,967 (81.54%)	1,647 (90.00%)	1,544 (84.42%)	1,463 (79.99%)	1,313 (71.75%)	
Yes	1,351 (18.46%)	183 (10.00%)	285 (15.58%)	366 (20.01%)	517 (28.25%)	
**Lung disease (%)**						0.003
No	6,459 (88.26%)	1,571 (85.85%)	1,634 (89.34%)	1,627 (88.96%)	1,627 (88.91%)	
Yes	859 (11.74%)	259 (14.15%)	195 (10.66%)	202 (11.04%)	203 (11.09%)	
**KOA (%)**						< 0.001
No	6,283 (85.86%)	1,620 (88.52%)	1,591 (86.99%)	1,572 (85.95%)	1,500 (81.97%)	
Yes	1,035 (14.14%)	210 (11.48%)	238 (13.01%)	257 (14.05%)	330 (18.03%)	

BRI, body roundness index; WBC, white blood cell; PLT, platelet; LDL-C, low-density lipoprotein cholesterol; BMI, body mass index; KOA, knee osteoarthritis.

### 3.2 Association between BRI and the risk of KOA

**Figure 2:**
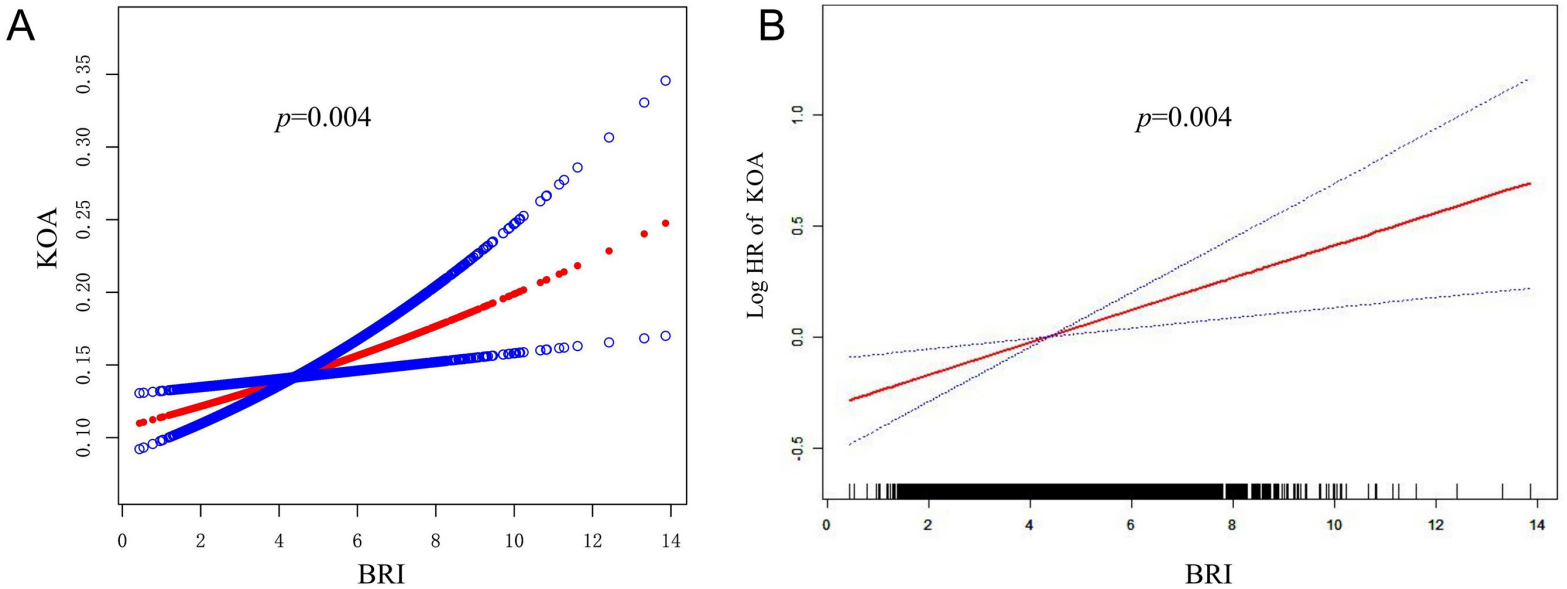
Association between BRI and KOA. **(A)** The red solid line represents the smooth curve fit between BRI and KOA. **(B)** The CI for the fit is represented by the red solid line band.

The study indicated a positive correlation between an increase in BRI and the risk of KOA (Table 2). This association was evident in the initial unadjusted Model 1 (*HR* = 1.15, 95% *CI*: 1.10–1.20, *p* < 0.0001) and remained significant in the subsequently adjusted Model 2 (*HR* = 1.08, 95% *CI*: 1.03–1.13; *p* = 0.0020) and the fully adjusted Model 3 (*HR* = 1.08, 95% *CI*: 1.02–1.13, *p* = 0.0039). The risk of KOA increased progressively with higher BRI quartiles (*p* for trend = 0.0033). Additionally, there is a linear correlation between smooth curve fits and weighted generalized additive models (Figure 2).

**Table 2:** Association between BRI and the risk of KOA.

	**Hazard ratio (95% CI)**, ***p*****-value**		
	**Model 1**	**Model 2**	**Model 3**
Continuous BRI	1.15 (1.10, 1.20) < 0.0001	1.08 (1.03, 1.13) 0.0020	1.08 (1.02, 1.13) 0.0039
**Categories BRI**			
Q1	Reference	Reference	Reference
Q2	1.15 (0.95, 1.41) 0.1563	1.08 (0.88, 1.31) 0.4804	1.12 (0.91, 1.37) 0.2752
Q3	1.26 (1.04, 1.53) 0.0197	1.12 (0.91, 1.37) 0.2763	1.16 (0.95, 1.43) 0.1518
Q4	1.70 (1.41, 2.05) < 0.0001	1.35 (1.11, 1.64) 0.0031	1.36 (1.11, 1.67) 0.0032
*p* for trend	1.19 (1.12, 1.26) < 0.0001	1.10 (1.03, 1.17) 0.0026	1.10 (1.03, 1.18) 0.0033

Model 1: non-adjusted; Model 2: adjusted age, sex, education, marital, residence; Model 3: adjusted age, sex, education, marital, residence, smoking, drinking, self-health, life-satisfaction, fall, stroke, diabetes, cardiovascular disease. BRI, body roundness index.

### 3.3 Evaluation of the prediction model

**Figure 3:**
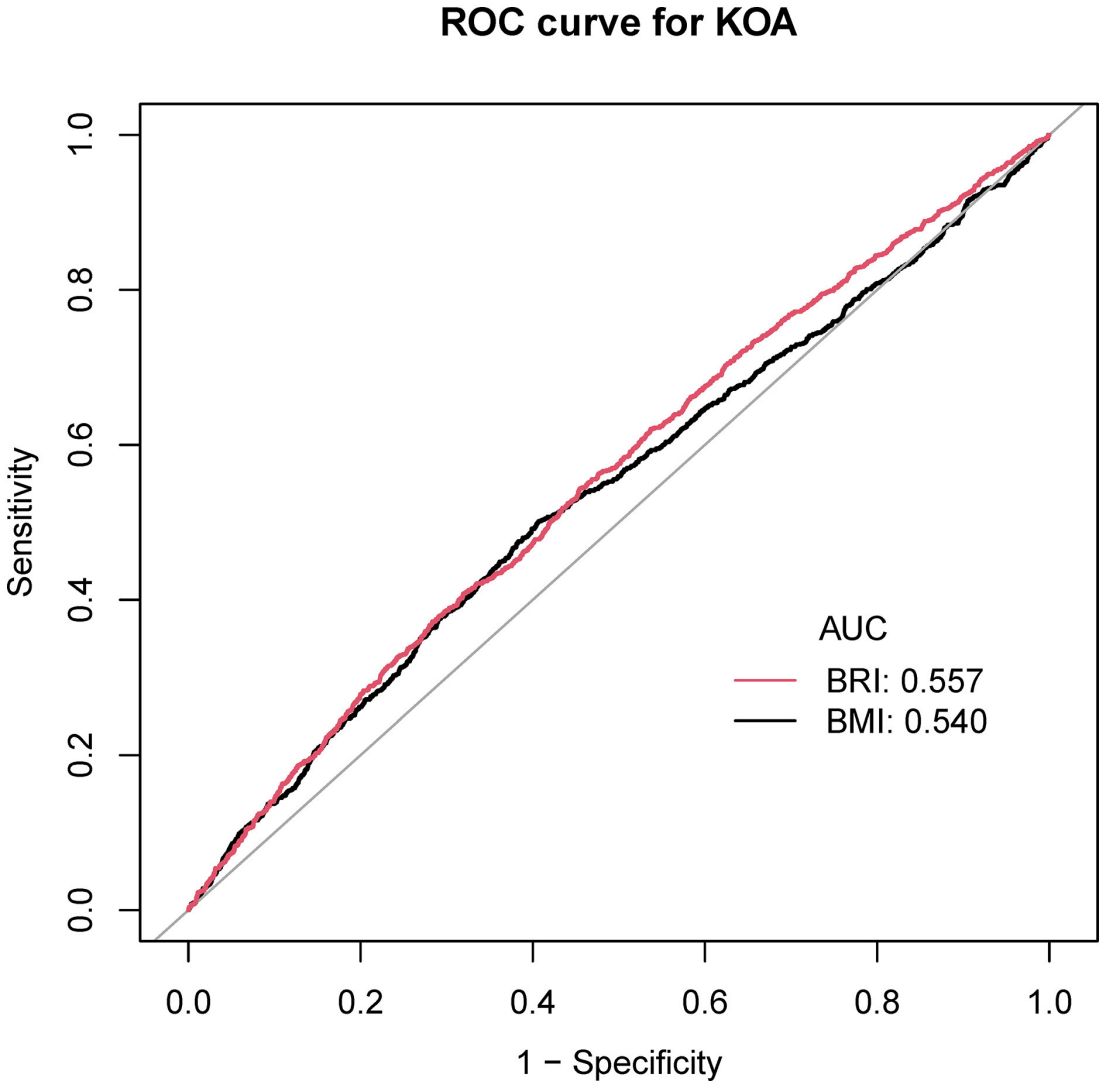
ROC curve for the evaluation of the prediction model. BRI, body roundness index; BMI, body mass index; KOA, knee osteoarthritis.

The area under the ROC curve was 0.557, indicating adequate discriminatory ability (95% CI: 0.538–0.576). Both BRI and BMI can predict the risk of KOA (area under the curve: 0.557 vs. 0.540, *p* = 0.0051; Figure 3 and Supplementary Table S1).

### 3.4 Subgroup analysis

**Figure 4:**
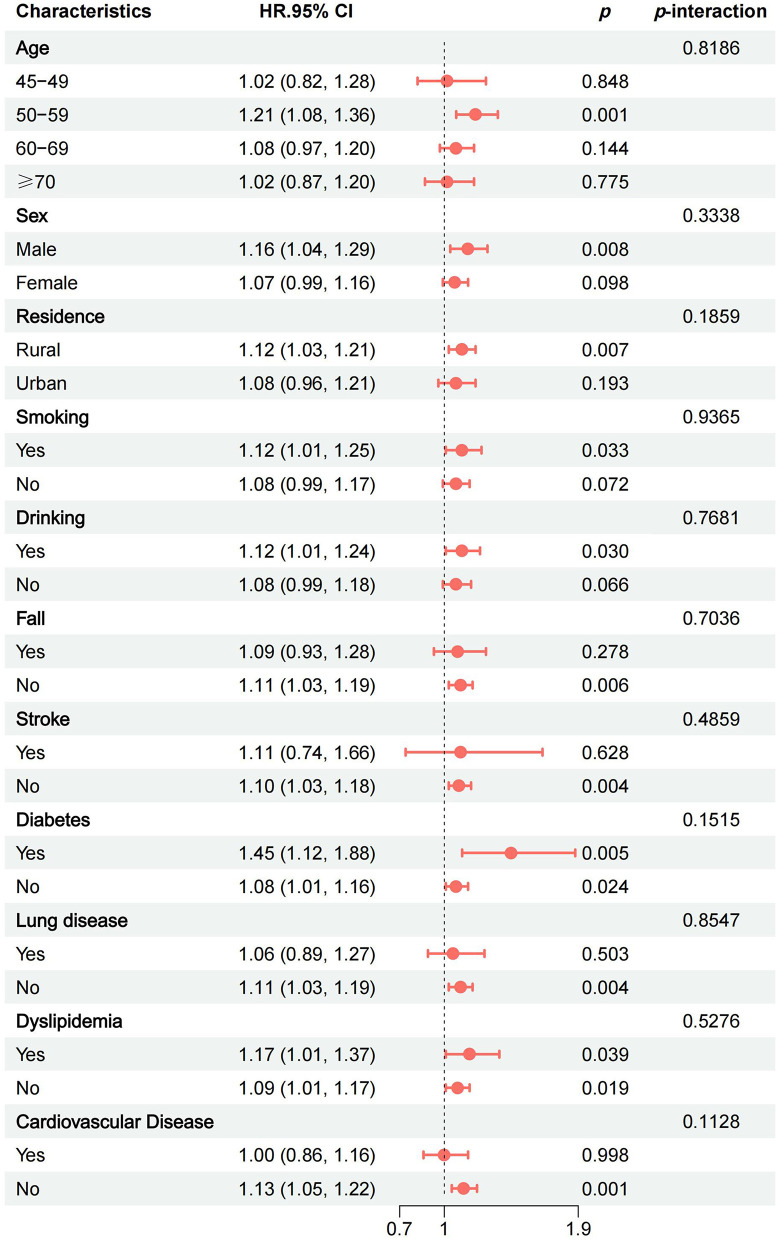
Forest plot of subgroup analysis of the association between BRI and the risk of KOA.

To control for potential variations in the positive correlation between BRI and KOA, we conducted subgroup analyses and interaction tests. The analyses were stratified by age, sex, residence, smoking, drinking, fall, stroke, diabetes, cardiovascular disease, dyslipidemia, and lung disease. Covariates adjusted in all subgroups included education, marital status, self-health, life-satisfaction, WBC, PLT, LDL-C, age, sex, residence, smoking, drinking, fall, stroke, diabetes, cardiovascular disease, and dyslipidemia. notably, in the age group of 50–59, an increase in BRI was associated with a heightened risk of KOA (*HR* = 1.21, 95% *CI*: 1.08–1.36, *p* = 0.001), as well as among males (*HR* = 1.12, 95% *CI*: 1.01–1.24, *p* = 0.008), those living in rural areas (*HR* = 1.12, 95% *CI*: 1.03–1.21, *p* = 0.007), smokers (*HR* = 1.09, 95% *CI*: 1.03–1.15, *p* = 0.033), drinkers (*HR* = 1.12, 95% *CI*: 1.01–1.24, *p* = 0.030), and participants without falls (*HR* = 1.11, 95% *CI*: 1.03–1.19, *p* = 0.006), without stroke (*HR* = 1.10, 95% *CI*: 1.03–1.18, *p*= 0.004), lung disease (*HR* = 1.11, 95% *CI*: 1.03–1.19, *p* = 0.004), and cardiovascular disease (*HR* = 1.10, 95% *CI*: 1.03–1.18, *p* = 0.004). In the interquartile subgroup analysis, compared with the Q1 group, the Q4 group had an increased risk of developing KOA in the age range of 50–59 years (*HR* = 1.79, 95% *CI*: 1.24–2.58, *p* = 0.002), male (*HR* = 1.46, 95% *CI*: 1.02–2.09, *p* = 0.039), those living in rural areas (*HR* = 1.46, 95% *CI*: 1.14–1.88, *p* = 0.003), drinkers (*HR* = 1.40, 95% *CI*: 1.01–1.94, *p* = 0.044), without falls (*HR* = 1.36, 95% *CI*: 1.09–1.71, *p* = 0.009), without stroke (*HR* = 1.34, 95% *CI*: 1.09–1.65, *p* = 0.006), cardiovascular disease (*HR* = 1.50, 95% *CI*: 1.19–1.88, *p* = 0.002), cardiovascular disease (*HR* = 1.50, 95% *CI*: 1.19–1.88, *p* = 0.002), non-dyslipidemia (*HR* = 1.34, 95% *CI*: 1.07–1.69, *p* = 0.012), and lung disease (*HR* = 1.40, 95% *CI*: 1.12–1.75, *p* = 0.003). However, interaction tests showed no significant interactions (all *p* > 0.05; Figure 4 and Supplementary Table S2).

## 4 Discussion

This longitudinal cohort study of 7,318 participants from the CHARLS database aimed to assess the association between BRI and KOA risk in the Chinese population. The results revealed a significant positive correlation between an increase in BRI and the prevalence of KOA, consistent across all adjusted models and subgroup analyses. Furthermore, our study found that, consistent with traditional BMI, BRI can serve as a discriminative index for KOA disease risk, indicating that BRI could be an effective tool for assessing KOA prevalence and offers valuable insights for early clinical prevention.

As a novel obesity measurement index, BRI has been widely employed in predicting the risks associated with various diseases due to its unique advantages (23, 24). First, similar to BMI's indication of fat accumulation, an increase in BRI also signifies enhanced body fat distribution (25), which may directly or indirectly impact the degeneration of weight-bearing joints such as the knee. High BRI values, indicative of increased adipose tissue content, are significant as adipose tissue acts as an active endocrine organ, secreting various pro-inflammatory factors like leptin, tumor necrosis factor-α, and interleukin-6. These factors potentially accelerate cartilage degeneration by inducing chondrocyte apoptosis or inhibiting their anabolic metabolism (26, 27). Additionally, adipose tissue metabolites, such as free fatty acids, may exacerbate joint inflammatory responses and oxidative stress, further degrading cartilage (28). Excessive adipose tissue can increase mechanical load on knee joints during exercise or daily activities, further exacerbating articular cartilage wear. In addition, the infrapatellar fat pad is an active tissue containing adipokines, cytokines, and chemokines. Under conditions of hypertrophy and mechanical pressure, the infrapatellar fat pad can release inflammatory factors, leading to local reactions and promoting mechanical and biological changes in the joint, increasing the inflammatory burden in the joint cavity and inducing KOA (29, 30). Moreover, visceral fat accumulation can also lead to synovial hyperplasia and immune system imbalances by enhancing inflammatory responses and altering the metabolic environment (31), all contributing significantly to KOA progression.

Compared to BMI, BRI not only reflects overall obesity levels but also captures the distribution characteristics of body fat more precisely, especially abdominal fat accumulation (11). The presence of abdominal fat is closely linked to the pathological process of KOA, as it is a major source of inflammatory factors and contributes to increased mechanical stress, thereby exacerbating joint degeneration (32). Therefore, BRI's role in predicting KOA risk may surpass that of BMI, particularly in identifying risks associated with systemic inflammation (33, 34). This longitudinal cohort study underscored a significant positive correlation between BRI and KOA risk, further highlighting the impact of body fat distribution in KOA progression. BRI's ability to reflect chronic low-grade systemic inflammation caused by visceral fat positions it as a crucial indicator in measuring body fat and understanding the progression of KOA. In addition, visceral fat may indirectly affect cartilage health and accelerate KOA progression by altering fatty acid metabolism and inducing insulin resistance (35). In addition, compared to BMI, BRI calculation requires more accurate body size data, which may lead to increased complexity and cost of measurement.

In our subgroup analysis, the risk association between BRI and KOA was more pronounced in men. Firstly, men tend to accumulate lipids primarily in the abdomen, resulting in increased visceral fat, which is closely linked to low-grade systemic inflammation (36). Excessive visceral fat leads to the secretion of numerous pro-inflammatory factors and heightened insulin resistance, exacerbating cartilage degeneration (37). Secondly, men often endure greater physical loads in daily and occupational activities, particularly in heavy manual labor roles. This excessive mechanical stress may further exacerbate knee joint wear. Finally, in men, behaviors such as smoking, consuming alcohol, and following high-fat diets are more prevalent. Smoking and alcohol use detrimentally impact articular cartilage by enhancing oxidative stress and promoting inflammatory factor release, while a high-fat diet increases body fat, especially visceral fat. These unhealthy lifestyles intensify the systemic inflammatory response, accelerating the onset and progression of KOA (38–40). Collectively, these factors underscore the more significant association between BRI and KOA in men. In addition, living in rural areas, where long-term engagement in heavy manual labor, limited rehabilitation and healthcare facilities, and poorer medical insurance coverage prevail, may explain the higher prevalence of KOA compared to urban settings (41). This includes being farther from high-quality food sources, having reduced access to walking facilities, fitness facilities, and professional medical care. Meanwhile, the population without falls, strokes, lung diseases, and cardiovascular diseases tends to have longer survival times, potentially engaging in more physical and social activities that could intensify cartilage damage. Conversely, those with falls, strokes, lung diseases, and cardiovascular diseases might have lower body weight due to the consumptive effects of chronic illnesses, which could indirectly reduce KOA risk. In subgroup analysis, it was also found that participants aged 50–59 had a higher risk of developing KOA, which may be related to increasing age, peak periods of work or family life, decreased estrogen levels in women during menopause, and weight gain in middle age (42).

Prior to this, although there have been reports about the positive correlation between BRI and KOA in the Chinese population (43), our research has further analyzed the correlation between BRI and KOA in different intervals, and further confirmed the correlation between BRI and KOA risk. However, our study also has some limitations. First, the BRI data were derived from body composition measurements, which, while relatively accurate in estimating body fat proportion, might still harbor measurement errors, particularly among the elderly. Secondly, since the sample predominantly came from specific areas, the generalizability of the results may be limited. Future studies should consider multi-center and large-sample validations. Thirdly, although this study provides important prospective data, the 5-year follow-up period may not be sufficient to fully capture the long-term relationship between BRI and KOA, particularly in older populations. Additionally, due to the fact that the study only collected data at a single time point and lacked a control group, this may affect the interpretability of the results. Moreover, although the relationship between BRI and KOA remained significant after adjusting for various confounding factors, the influence of potential unknown confounding factors cannot be overlooked.

## 5 Conclusion

This study further supports the use of BRI as a tool for KOA risk assessment. These findings have significant clinical implications for the early prevention and management of KOA, particularly in personalized interventions targeting body fat distribution. Future research should further explore the combined role of BRI and other body composition indicators in predicting KOA, especially how to more accurately identify high-risk individuals through the combination of multiple indicators and implement targeted prevention and treatment interventions.

## Data Availability

The datasets presented in this study can be found in online repositories. The names of the repository/repositories and accession number(s) can be found in the article/Supplementary material.

## References

[1] Turkiewicz A Gerhardsson DVM Engstrom G Nilsson PM Mellstrom C Lohmander LS. Prevalence of knee pain and knee OA in southern Sweden and the proportion that seeks medical care. Rheumatology. (2015) 54:827–35. 10.1093/rheumatology/keu409 25313145

[2] Yu H Huang T Lu WW Tong L Chen D. Osteoarthritis pain. Int J Mol Sci. (2022) 23:4642. 10.3390/ijms23094642 35563035 PMC9105801

[3] Du X Liu ZY Tao XX Mei YL Zhou DQ Cheng K. Research progress on the pathogenesis of knee osteoarthritis. Orthop Surg. (2023) 15:2213–24. 10.1111/os.13809 37435789 PMC10475681

[4] Cui A Li H Wang D Zhong J Chen Y Lu H. Global, regional prevalence, incidence and risk factors of knee osteoarthritis in population-based studies. Eclinicalmedicine. (2020) 29–30:100587. 10.1016/j.eclinm.2020.100587 34505846 PMC7704420

[5] Scanzello CR Goldring SR. The role of synovitis in osteoarthritis pathogenesis. Bone. (2012) 51:249–57. 10.1016/j.bone.2012.02.012 22387238 PMC3372675

[6] Olivotto E Trisolino G Belluzzi E Lazzaro A Strazzari A Pozzuoli A. Macroscopic synovial inflammation correlates with symptoms and cartilage lesions in patients undergoing arthroscopic partial meniscectomy: a clinical study. J Clin Med. (2022) 11:4330. 10.3390/jcm11154330 35893418 PMC9330366

[7] Long H Zeng X Liu Q Wang H Vos T Hou Y. Burden of osteoarthritis in China, 1990-2017: findings from the Global Burden of Disease Study 2017. Lancet Rheumatol. (2020) 2:e164–72. 10.1016/S2665-9913(19)30145-6 38263654

[8] Ren Y Hu J Tan J Tang X Li Q Yang H. Incidence and risk factors of symptomatic knee osteoarthritis among the Chinese population: analysis from a nationwide longitudinal study. BMC Public Health. (2020) 20:1491. 10.1186/s12889-020-09611-7 33004017 PMC7528331

[9] Mobasheri A Thudium CS Bay-Jensen AC Maleitzke T Geissler S Duda GN. Biomarkers for osteoarthritis: current status and future prospects. Best Pract Res Clin Rheumatol. (2023) 37:101852. 10.1016/j.berh.2023.101852 37620236

[10] Gambari L Cellamare A Grassi F Grigolo B Panciera A Ruffilli A. Targeting the inflammatory hallmarks of obesity-associated osteoarthritis: towards nutraceutical-oriented preventive and complementary therapeutic strategies based on n-3 polyunsaturated fatty acids. Int J Mol Sci. (2023) 24:9340. 10.3390/ijms24119340 37298291 PMC10253881

[11] Thomas DM Bredlau C Bosy-Westphal A Mueller M Shen W Gallagher D. Relationships between body roundness with body fat and visceral adipose tissue emerging from a new geometrical model. Obesity. (2013) 21:2264–71. 10.1002/oby.20408 23519954 PMC3692604

[12] Yang M Liu J Shen Q Chen H Liu Y Wang N. Body roundness index trajectories and the incidence of cardiovascular disease: evidence from the China health and retirement longitudinal study. J Am Heart Assoc. (2024) 13:e34768. 10.1161/JAHA.124.034768 39319466 PMC11681446

[13] Rico-Martin S Calderon-Garcia JF Sanchez-Rey P Franco-Antonio C Martinez AM Sanchez MJ. Effectiveness of body roundness index in predicting metabolic syndrome: a systematic review and meta-analysis. Obes Rev. (2020) 21:e13023. 10.1111/obr.13023 32267621

[14] Feng J He S Chen X. Body adiposity index and body roundness index in identifying insulin resistance among adults without diabetes. Am J Med Sci. (2019) 357:116–23. 10.1016/j.amjms.2018.11.006 30665492

[15] Jansen N Molendijk E Schiphof D van Meurs J Oei E van Middelkoop M. Metabolic syndrome and the progression of knee osteoarthritis on MRI. Osteoarthritis Cartilage. (2023) 31:647–55. 10.1016/j.joca.2023.02.003 36801367

[16] Kuusalo L Felson DT Wang N Lewis CE Torner J Nevitt MC. Metabolic osteoarthritis - relation of diabetes and cardiovascular disease with knee osteoarthritis. Osteoarthritis Cartilage. (2021) 29:230–34. 10.1016/j.joca.2020.09.010 33253888 PMC8020447

[17] Shumnalieva R Kotov G Ermencheva P Monov S. Pathogenic mechanisms and therapeutic approaches in obesity-related knee osteoarthritis. Biomedicines. (2023) 12:9. 10.3390/biomedicines12010009 38275369 PMC10812969

[18] Wang X Guo Z Wang M Xiang C. Association between body roundness index and risk of osteoarthritis: a cross-sectional study. Lipids Health Dis. (2024) 23:334. 10.1186/s12944-024-02324-5 39402634 PMC11472493

[19] Ke T Lai J Li X Liu F Liu W Zhong C. Association between the body roundness index and osteoarthritis: evidence from NHANES. Front Med. (2024) 11:1472196. 10.3389/fmed.2024.1472196 39512614 PMC11540616

[20] Liang H Si W Li L Yang K. Association between body roundness index and osteoarthritis: a cross-sectional analysis of NHANES 2011-2018. Front Nutr. (2024) 11:1501722. 10.3389/fnut.2024.1501722 39545042 PMC11560466

[21] Zhao Y Hu Y Smith JP Strauss J Yang G. Cohort profile: the China Health and Retirement Longitudinal Study (CHARLS). Int J Epidemiol. (2014) 43:61–8. 10.1093/ije/dys203 23243115 PMC3937970

[22] Li W Liu E Balezentis T Jin H Streimikiene D. Association between socioeconomic welfare and depression among older adults: evidence from the China health and Retirement Longitudinal Study. Soc Sci Med. (2021) 275:113814. 10.1016/j.socscimed.2021.113814 33721747

[23] Gao W Jin L Li D Zhang Y Zhao W Zhao Y. The association between the body roundness index and the risk of colorectal cancer: a cross-sectional study. Lipids Health Dis. (2023) 22:53. 10.1186/s12944-023-01814-2 37072848 PMC10111650

[24] Zhang L Yin J Sun H Dong W Liu Z Yang J. The relationship between body roundness index and depression: a cross-sectional study using data from the National Health and Nutrition Examination Survey (NHANES) 2011-2018. J Affect Disord. (2024) 361:17–23. 10.1016/j.jad.2024.05.153 38815765

[25] Ching YK Chin YS Appukutty M Gan WY Chan YM. Comparisons of conventional and novel anthropometric obesity indices to predict metabolic syndrome among vegetarians in Malaysia. Sci Rep. (2020) 10:20861. 10.1038/s41598-020-78035-5 33257810 PMC7705716

[26] Coppack SW. Pro-inflammatory cytokines and adipose tissue. Proc Nutr Soc. (2001) 60:349–56. 10.1079/PNS2001110 11681809

[27] Xie C Chen Q. Adipokines: new therapeutic target for osteoarthritis? Curr Rheumatol Rep. (2019) 21:71. 10.1007/s11926-019-0868-z 31813080 PMC7291783

[28] Courties A Gualillo O Berenbaum F Sellam J. Metabolic stress-induced joint inflammation and osteoarthritis. Osteoarthritis Cartilage. (2015) 23:1955–65. 10.1016/j.joca.2015.05.016 26033164

[29] Fontanella CG Belluzzi E Pozzuoli A Scioni M Olivotto E Reale D. Exploring anatomo-morphometric characteristics of infrapatellar, suprapatellar fat pad, and knee ligaments in osteoarthritis compared to post-traumatic lesions. Biomedicines. (2022) 10:1369. 10.3390/biomedicines10061369 35740391 PMC9220326

[30] Zeng N Yan ZP Chen XY Ni GX. Infrapatellar fat pad and knee osteoarthritis. Aging Dis. (2020) 11:1317–28. 10.14336/AD.2019.1116 33014539 PMC7505265

[31] Binvignat M Sellam J Berenbaum F Felson DT. The role of obesity and adipose tissue dysfunction in osteoarthritis pain. Nat Rev Rheumatol. (2024) 20:565–84. 10.1038/s41584-024-01143-3 39112603

[32] Urban H Little CB. The role of fat and inflammation in the pathogenesis and management of osteoarthritis. Rheumatology. (2018) 57:v10–21. 10.1093/rheumatology/kex399 29444323

[33] Chen Y Wang C Sun Q Ye Q Zhou H Qin Z. Comparison of novel and traditional anthropometric indices in Eastern-China adults: which is the best indicator of the metabolically obese normal weight phenotype? BMC Public Health. (2024) 24:2192. 10.1186/s12889-024-19638-9 39138449 PMC11321156

[34] Mirzababaei A Abaj F Khosravinia D Ghorbani M Valisoltani N Clark C. The mediatory effect of inflammatory markers on the association between a body shape index and body roundness index with cardiometabolic risk factor in overweight and obese women: a cross-sectional study. Front Nutr. (2023) 10:1178829. 10.3389/fnut.2023.1178829 37360300 PMC10288880

[35] Tchetina EV Markova GA Sharapova EP. Insulin resistance in osteoarthritis: similar mechanisms to type 2 diabetes mellitus. J Nutr Metab. (2020) 2020:4143802. 10.1155/2020/4143802 32566279 PMC7261331

[36] Ouchi N Parker JL Lugus JJ Walsh K. Adipokines in inflammation and metabolic disease. Nat Rev Immunol. (2011) 11:85–97. 10.1038/nri2921 21252989 PMC3518031

[37] Sun AR Udduttula A Li J Liu Y Ren PG Zhang P. Cartilage tissue engineering for obesity-induced osteoarthritis: physiology, challenges, and future prospects. J Orthop Translat. (2021) 26:3–15. 10.1016/j.jot.2020.07.004 33437618 PMC7773977

[38] Fernandez-Torres J Zamudio-Cuevas Y Martinez-Nava GA Aztatzi-Aguilar OG Sierra-Vargas MP Lozada-Perez CA. Correction to: impact of cadmium mediated by tobacco use in musculoskeletal diseases. Biol Trace Elem Res. (2022) 200:2016. 10.1007/s12011-021-02853-5 34345954

[39] Wang J Zhang B Peng L Wang J Xu K Xu P. The Causal association between alcohol, smoking, coffee consumption, and the risk of arthritis: a meta-analysis of Mendelian randomization studies. Nutrients. (2023) 15:5009. 10.3390/nu15235009 38068867 PMC10707754

[40] Jiang W Chen H Lin Y Cheng K Zhou D Chen R. Mechanical stress abnormalities promote chondrocyte senescence - the pathogenesis of knee osteoarthritis. Biomed Pharmacother. (2023) 167:115552. 10.1016/j.biopha.2023.115552 37748410

[41] Messier SP Monroe MG Callahan LF Mihalko SL Beavers DP Queen K. Disparities between rural and urban communities: response to 18 months of diet and exercise versus control for knee osteoarthritis and overweight or obesity. Arthritis Care Res. (2024). 10.1002/acr.25448 39400996 PMC11684979

[42] Stevens-Lapsley JE Kohrt WM. Osteoarthritis in women: effects of estrogen, obesity and physical activity. Womens Health. (2010) 6:601–15. 10.2217/WHE.10.38 20597623

[43] Lv H Wang Y Zhang G Wang X Hu Z Chu Q. Association between obesity measurement indexes and symptomatic knee osteoarthritis among the Chinese population: analysis from a nationwide longitudinal study. BMC Musculoskelet Disord. (2024) 25:986. 10.1186/s12891-024-08009-5 39623424 PMC11610057

