# Supplementary material for: Body roundness index and the risk of knee osteoarthritis: evidence from the China Health and Retirement Longitudinal Study
Source: Front Nutr. 2025 Mar 12;12:1533966. doi: 10.3389/fnut.2025.1533966 (PMC11938369; doi:10.3389/fnut.2025.1533966)
Supplement: Supplementary file 1 [file Table_1.docx]

Table S1 ROC analysis for continuous predictor

| **Test** | **KOA** | **Non KOA** | **ROC area(AUC)** | **95%CI low** | **95%CI upp** | **Best threshold** | **Specificity** | **Sensitivity** | ***p*-value (vs BMI)** |
| --- | --- | --- | --- | --- | --- | --- | --- | --- | --- |
| BRI | 1035 | 6283 | 0.557 | 0.5379 | 0.5761 | 4.3719 | 0.545 | 0.544 | 0.0051 |
| BMI | 1035 | 6283 | 0.5403 | 0.5206 | 0.56 | 24.4886 | 0.5932 | 0.5014 | - |

BRI indicates the body roundness index; BMI indicates the body mass index; KOA, knee osteoarthritis.

Table S2 Subgroup analysis between BRI and KOA risks

| **Characteristics** | | **BRI, Hazard Ratio (95% CI), *P*-value** | **BRI quartile, Hazard Ratio (95% CI), *P*-value** | | | |
| --- | --- | --- | --- | --- | --- | --- |
|  |  |  | Q1 | Q2 | Q3 | Q4 |
| **Age** | 45-49 | 0.99 (0.81, 1.20) 0.894 | Reference | 0.96 (0.47, 1.95) 0.9121 | 1.18 (0.59, 2.37) 0.6324 | 1.00 (0.49, 2.07) 0.992 |
|  | 50-59 | 1.15 (1.05, 1.25) 0.003 | Reference | 1.22 (0.85, 1.75) 0.2767 | 1.46 (1.02, 2.10) 0.0402 | 1.79 (1.24, 2.58) 0.002 |
|  | 60-69 | 1.07 (0.99, 1.15) 0.107 | Reference | 1.33 (0.97, 1.83) 0.0807 | 1.11 (0.80, 1.55) 0.5173 | 1.37 (0.99, 1.89) 0.058 |
|  | ≥70 | 1.03 (0.92, 1.16) 0.563 | Reference | 0.78 (0.47, 1.29) 0.3345 | 0.89 (0.54, 1.47) 0.6450 | 1.02 (0.62, 1.67) 0.937 |
| **Sex** | Female | 1.05 (0.99, 1.12) 0.090 | Reference | 1.03 (0.77, 1.36) 0.8617 | 0.96 (0.73, 1.27) 0.8007 | 1.22 (0.94, 1.60) 0.133 |
|  | Male | 1.14 (1.04, 1.25) 0.007 | Reference | 1.20 (0.89, 1.61) 0.2339 | 1.50 (1.10, 2.04) 0.0098 | 1.46 (1.02, 2.09) 0.039 |
| **Residence** | Urban | 1.02 (0.94, 1.12) 0.578 | Reference | 0.97 (0.66, 1.42) 0.8778 | 1.18 (0.83, 1.69) 0.3506 | 1.20 (0.83, 1.71) 0.329 |
|  | Rural | 1.11 (1.04, 1.18) 0.001 | Reference | 1.19 (0.93, 1.52) 0.1641 | 1.12 (0.87, 1.44) 0.3941 | 1.46 (1.14, 1.88) 0.003 |
| **Smoking** | No | 1.06 (1.00, 1.13) 0.060 | Reference | 1.06 (0.80, 1.41) 0.6989 | 1.01 (0.77, 1.34) 0.9314 | 1.26 (0.96, 1.65) 0.090 |
|  | Yes | 1.10 (1.00, 1.20) 0.042 | Reference | 1.18 (0.88, 1.59) 0.2787 | 1.39 (1.02, 1.89) 0.0378 | 1.36 (0.96, 1.93) 0.083 |
| **Drinking** | No | 1.07 (1.00, 1.13) 0.046 | Reference | 1.07 (0.81, 1.42) 0.6157 | 1.03 (0.78, 1.37) 0.8112 | 1.28 (0.98, 1.68) 0.067 |
|  | Yes | 1.08 (1.00, 1.18) 0.059 | Reference | 1.14 (0.84, 1.54) 0.3989 | 1.29 (0.95, 1.75) 0.0983 | 1.40 (1.01, 1.94) 0.044 |
| **Fall** | No | 1.09 (1.03, 1.15) 0.003 | Reference | 1.12 (0.90, 1.41) 0.3099 | 1.20 (0.96, 1.51) 0.1049 | 1.36 (1.09, 1.71) 0.007 |
|  | Yes | 1.04 (0.93, 1.17) 0.505 | Reference | 1.08 (0.66, 1.78) 0.7538 | 0.97 (0.58, 1.62) 0.9068 | 1.34 (0.82, 2.20) 0.243 |
| **Stroke** | No | 1.07 (1.02, 1.13) 0.006 | Reference | 1.09 (0.88, 1.34) 0.4215 | 1.17 (0.95, 1.44) 0.1502 | 1.34 (1.09, 1.65) 0.006 |
|  | Yes | 1.17 (0.86, 1.60) 0.309 | Reference | 2.30 (0.62, 8.60) 0.2156 | 0.79 (0.18, 3.35) 0.7439 | 1.96 (0.53, 7.33) 0.315 |
| **Diabetes** | No | 1.06 (1.00, 1.12) 0.037 | Reference | 1.11 (0.90, 1.36) 0.3453 | 1.14 (0.92, 1.40) 0.2381 | 1.28 (1.04, 1.59) 0.022 |
|  | Yes | 1.24 (1.06, 1.45) 0.008 | Reference | 1.62 (0.56, 4.66) 0.3690 | 2.14 (0.77, 5.93) 0.1451 | 3.22 (1.22, 8.54) 0.019 |
| **Cardiovascular Disease** | No | 1.10 (1.04, 1.17) 0.001 | Reference | 1.19 (0.95, 1.48) 0.1354 | 1.19 (0.95, 1.49) 0.1312 | 1.50 (1.19, 1.88) 0.001 |
|  | Yes | 1.00 (0.90, 1.11) 0.994 | Reference | 0.90 (0.54, 1.52) 0.6991 | 0.96 (0.58, 1.59) 0.8757 | 0.96 (0.59, 1.57) 0.876 |
| **Dyslipidemia** | No | 1.08 (1.02, 1.14) 0.007 | Reference | 1.19 (0.96, 1.49) 0.1149 | 1.18 (0.94, 1.48) 0.1514 | 1.34 (1.07, 1.69) 0.012 |
|  | Yes | 1.07 (0.96, 1.20) 0.193 | Reference | 0.79 (0.44, 1.40) 0.4142 | 1.10 (0.65, 1.86) 0.7272 | 1.36 (0.83, 2.24) 0.225 |
| **Lung disease** | No | 1.08 (1.02, 1.14) 0.005 | Reference | 1.15 (0.92, 1.44) 0.2129 | 1.13 (0.91, 1.42) 0.2705 | 1.40 (1.12, 1.75) 0.003 |
|  | Yes | 1.04 (0.91, 1.19) 0.526 | Reference | 0.96 (0.55, 1.67) 0.8929 | 1.29 (0.77, 2.17) 0.3333 | 1.12 (0.64, 1.94) 0.697 |

Adjusted: Education; Marital; Self-health; Life-satisfaction; WBC; PLT; LDL-C; Age; Sex; Residence; Smoking; Drinking; Fall; Stroke; Diabetes; Cardiovascular Disease; Dyslipidemia. BRI indicates the body roundness index; KOA, knee osteoarthritis.

(HR=1.79, 95% CI: 1.24-2.58, p=0.002),
